# Effects of nutritional components on aging

**DOI:** 10.1111/acel.12277

**Published:** 2014-10-22

**Authors:** Dongyeop Lee, Wooseon Hwang, Murat Artan, Dae-Eun Jeong, Seung-Jae Lee

**Affiliations:** 1 Department of Life Sciences, Pohang University of Science and TechnologyPohang, Gyeongbuk, South Korea; 2 Information Technology Convergence Engineering, Pohang University of Science and TechnologyPohang, Gyeongbuk, South Korea; 3 School of Interdisciplinary Bioscience and Bioengineering, Pohang University of Science and TechnologyPohang, Gyeongbuk, 790-784, South Korea

**Keywords:** aging, amino acid, carbohydrate, lipid, mineral, nutrient, protein, vitamin

## Abstract

Nutrients including carbohydrates, proteins, lipids, vitamins, and minerals regulate various physiological processes and are essential for the survival of organisms. Reduced overall caloric intake delays aging in various organisms. However, the role of each nutritional component in the regulation of lifespan is not well established. In this review, we describe recent studies focused on the regulatory role of each type of nutrient in aging. Moreover, we will discuss how the amount or composition of each nutritional component may influence longevity or health in humans.

## Introduction

All organisms must obtain nutrients from their environments to live. These nutrients include organic chemicals, such as carbohydrates, proteins, lipids, and vitamins, and inorganic substances, such as minerals and water. The nutrients are essential for the maintenance of biological functions, including metabolism, growth, and repair. Interestingly, calorie restriction (CR), which is defined as a reduced intake of nutritional calories without malnutrition, has been shown to enhance the maintenance of biological systems and to increase lifespan (Kenyon, 2010). The molecular signaling pathways that mediate the effects of CR on longevity have been actively studied. In addition, many studies have specified which nutritional components contribute to aging, including early mammalian studies (Maeda *et al*., 1985; Masoro, 1985, 2002; Yu *et al*., 1985; Iwasaki *et al*., 1988a,b; Weindruch & Walford, 1988; Masoro *et al*., 1989), and this is currently an active area of investigation. Here, we will review recent findings regarding the effects of major dietary nutrients, namely carbohydrates, proteins and amino acids, lipids, and vitamins and minerals, on the lifespan of a diverse group of organisms, ranging from yeast to mammals. In addition to several excellent reviews on this topic (Piper *et al*., 2005, 2011; Tatar *et al*., 2014), our current review covers comprehensive ranges of nutritional components and their effects on aging in various organisms. Further, there is now increased understanding of the importance of diet for aging and age-related diseases in humans. Therefore, we will discuss the potential influences of these dietary components on human aging and age-related diseases.

## Effects of carbohydrates on aging

**Figure 1:**
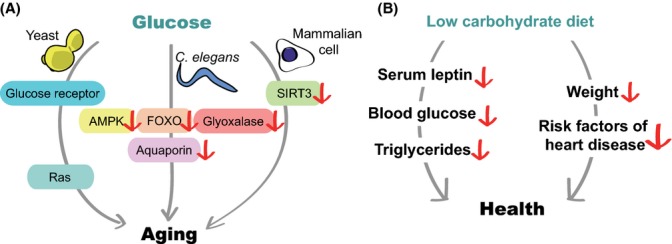
Glucose accelerates aging through various signaling pathways. (A) A high-glucose diet shortens lifespan in various organisms. In yeast, glucose decreases lifespan through the glucose receptor and Ras, components of a growth-promoting signaling pathway. In *Caenorhabditis elegans*, glucose downregulates pro-longevity proteins, such as AMP-activated protein kinase (AMPK), FOXO, and glyoxalase, resulting in short lifespan. Sirtuin 3 (SIRT3), an NAD-dependent protein deacetylase, mediates the effects of glucose on senescence in cultured mammalian cells. (B) Low-carbohydrate diets may improve human health by reducing several factors, including serum leptin, blood glucose, and triglycerides, which are associated with aging or metabolic defects. In addition, reduced carbohydrate intake decreases body weight and reduces the risk factors associated with heart disease.

Carbohydrates are organic compounds comprised of carbon, hydrogen, and oxygen. Carbohydrates act as signaling molecules, energy sources, and structural components. The importance of carbohydrates for human health is exemplified by the tight association between chronic metabolic diseases and carbohydrate-rich diets. Such diets have high glycemic indices, which result in rapid increases in blood glucose levels (Jenkins *et al*., 1981). In addition, recent studies indicate that several dietary carbohydrates directly influence lifespan in various organisms through diverse signaling pathways (Fig. 1).

### Glucose alters lifespan through energy-sensing signaling pathways

Glucose, the primary energy source of most living organisms, is one of the best-studied carbohydrates that affect aging. Increased glucose intake accelerates aging in several model organisms, including yeast and *Caenorhabditis elegans*. Glucose-enriched diets shorten the lifespan of *C. elegans* by downregulating the activity of pro-longevity proteins, including AMP-activated protein kinase (AMPK), a FOXO transcription factor, and glyoxalase (Schulz *et al*., 2007; Lee *et al*., 2009; Schlotterer *et al*., 2009). Glucose consumption decreases the activity of AMPK, an energy sensor that regulates organismal lifespan. In contrast, treatment with a glucose analog, 2-deoxy-glucose, leads to glucose restriction, activation of AMPK, and longevity (Schulz *et al*., 2007). Glucose consumption decreases the activity of FOXO, a key downstream longevity transcription factor in the insulin/insulin-like growth factor-1 (IGF-1) signaling pathway (Lee *et al*., 2009). Reduced FOXO activity downregulates the aquaporin-1/glycerol channel and alters glycerol levels to shorten lifespan (Lee *et al*., 2009). Glucose-enriched diets also increase the level of methylglyoxal, a toxic advanced glycation end-product that is generated by nonenzymatic reactions during glucose metabolism, and this in turn reduces lifespan (Schlotterer *et al*., 2009). Moreover, recent studies show that the effect of glucose on the lifespan of *C. elegans* is modulated by a glucose transporter (Feng *et al*., 2013; Kitaoka *et al*., 2013) and pro-apoptotic genes (Choi, 2011). Thus, high dietary glucose appears to decrease the lifespan of *C. elegans* by influencing the activity of a variety of proteins that regulate lifespan and metabolism. The mechanisms through which glucose affects these factors coordinately or individually remain unclear.

Amounts of glucose negatively correlate with the lifespan of budding and fission yeasts (Roux *et al*., 2009; Weinberger *et al*., 2010). Glucose restriction, which is similar to dietary restriction (DR), increases the lifespan of the budding yeast *Saccharomyces cerevisiae*. However, excess glucose decreases lifespan through growth-promoting signaling proteins, such as Sch9, Tor1, and Ras (Weinberger *et al*., 2010). The glucose-sensing G-protein-coupled receptor Git3p (Welton & Hoffman, 2000) mediates the lifespan-shortening effect of glucose in the fission yeast *Schizosaccharomyces pombe* (Roux *et al*., 2009). Based on additional genetic data, glucose was proposed to activate Git3p, reducing lifespan through the activation of Gα and downstream Ras-cAMP/PKA signaling (Roux *et al*., 2009). Thus, the Ras pathway, one of the first signaling pathways to be implicated in the regulation of yeast lifespan (Chen *et al*., 1990), appears to play a central role in the effect of glucose on lifespan.

Glucose may also accelerate aging in mammals, although direct evidence is scarce. High concentrations of glucose in media accelerate the senescence of cultured human cells (Mortuza *et al*., 2013; Zhang *et al*., 2013). This pro-aging effect of glucose is associated with reduced expression of sirtuins, including SIRT3/sirtuin 3 (Mortuza *et al*., 2013; Zhang *et al*., 2013), a nicotinamide adenine dinucleotide (NAD)-dependent protein deacetylase (Haigis & Guarente, 2006). In addition, shRNA-mediated knockdown of SIRT3 accelerates senescence, whereas overexpression of SIRT3 suppresses glucose-induced cellular senescence (Zhang *et al*., 2013). Because glycolysis consumes NAD to produce NADH, the high-energy state caused by excess glucose may accelerate cellular senescence by downregulating the activity of sirtuins, such as SIRT3 (Kassi & Papavassiliou, 2008). It will be interesting to examine genetic mouse models of *SIRT3* to determine whether this finding is consistent with organismal aging.

### Carbohydrates that extend lifespan

In contrast to glucose, several other carbohydrates or carbohydrate metabolites, including trehalose, pyruvate, malate, fumarate, and *N*-acetylglucosamine (GlcNAc), have been shown to promote longevity in *C. elegans* (Honda *et al*., 2010; Mouchiroud *et al*., 2011; Edwards *et al*., 2013; Denzel *et al*., 2014). In particular, it is intriguing that a disaccharide trehalose is linked to longevity in yeast and *C. elegans* (Honda *et al*., 2010; Trevisol *et al*., 2011), because its monomer glucose decreases lifespan as described above. Trehalose feeding also increases stress resistance in *C. elegans*, which is consistent with the ability of trehalose to protect invertebrates from various stresses (Honda *et al*., 2010). Moreover, mutations that cause accumulation of trehalose promote fermentative capacity and extend the lifespan of yeast (Trevisol *et al*., 2011). Thus, trehalose appears to increase lifespan by acting as a general antistress sugar in invertebrates. In addition, GlcNAc, which is generated from glucose, increases the lifespan of *C. elegans* by improving the homeostasis of endoplasmic reticulum (ER) proteins (Denzel *et al*., 2014). Thus, trehalose and GlcNAc, which are metabolites of life-shortening glucose, appear to exert beneficial effects on lifespan in *C. elegans*.

### Variable effects of carbohydrates on different model organisms

The effects of carbohydrates on aging are variable depending on species. In flies, the ratio of protein and carbohydrate (P:C) appears more important for lifespan regulation than individual nutrients (Mair *et al*., 2005; Min & Tatar, 2006; Lee *et al*., 2008; Skorupa *et al*., 2008; Fanson *et al*., 2009; Bruce *et al*., 2013). Likewise, low P:C diets are beneficial for health and aging in rodents (Solon-Biet *et al*., 2014). These studies point to crucial roles of proteins in lifespan regulation in flies and mammals (see the next section). Why are there differences among different species? First of all, we have to consider the possibility that different species have distinct physiological responses or diet-responsive signaling pathways to ingested nutrients depending on ecology. For example, responses to sugars may be more crucial for worms, but proteins are more important for flies in their natural habitats. Second, glucose is the most commonly used dietary carbohydrate for culturing *C. elegans* and yeast, whereas sucrose is used for *Drosophila* and rodents. In addition, studies using diets with defined nutrient composition are scarce in invertebrate models, because in most experimental paradigms, worms and flies, respectively, feed on *Escherichia coli* and yeast, which contain complex nutrients. Thus, future studies equipped with better understanding of the ecology of organisms and with defined diet systems will be crucial for addressing these questions.

### Potential roles for dietary carbohydrates in human aging

Although the roles of dietary carbohydrates in human aging are unclear, clinical studies show that a low-carbohydrate diet is beneficial for human health (Rosedale *et al*., 2009). Administration of low-carbohydrate diets with high amounts of fats and adequate quantities of proteins significantly reduces body weight after 3 months (Rosedale *et al*., 2009). In addition, the human subjects show decreased levels of serum leptin, insulin, fasting glucose, and triglycerides, which are implicated in aging and metabolic defects. Low-carbohydrate diets also reduce body weight and several risk factors for heart disease (Foster *et al*., 2003). Conversely, high glycemic load diets enriched with carbohydrates positively correlate with age-related diseases including diabetes and heart diseases (Aston, 2006; Barclay *et al*., 2008). Thus, a low-carbohydrate diet may delay aging in humans by preventing metabolic diseases and improving general health.

## Effects of dietary proteins and amino acids on aging

**Figure 2:**
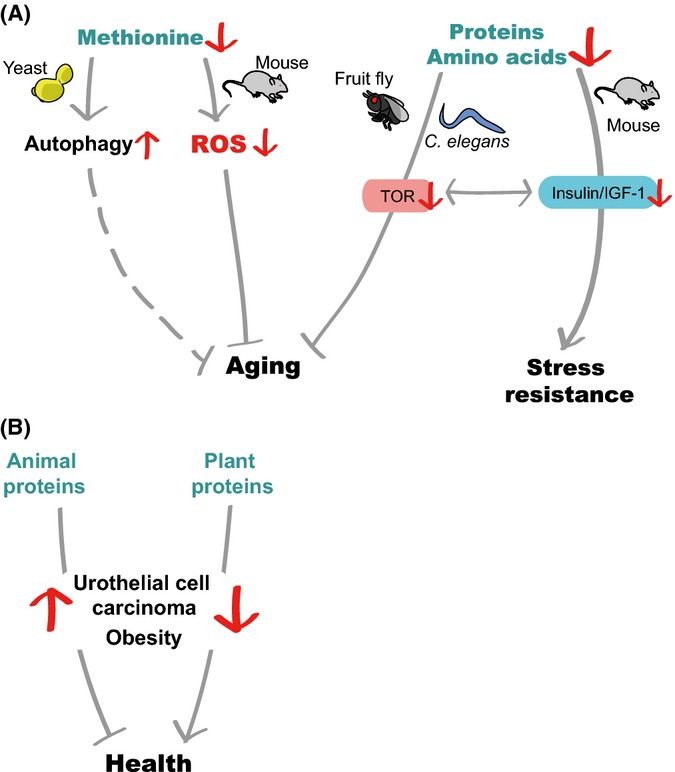
Restriction of amino acids or proteins increases stress resistance and influences longevity in various model organisms. (A) In yeast, methionine restriction decreases translational capacity and increases autophagy, indirectly interfering with aging. In addition, methionine restriction increases the lifespan of mice, possibly by downregulating the levels of reactive oxygen species (ROS). Dietary restrictions of protein intake increase the lifespan of various insects, including *Drosophila*. In *Caenorhabditis elegans*, reduced protein intake extends lifespan by inhibiting the insulin/insulin-like growth factor-1 (IGF-1) and target of rapamycin (TOR) signaling pathways. In mice, restrictions on the intake of protein or specific amino acids decrease oxidative stress by reducing insulin/IGF-1 signaling. (B) The levels of animal proteins positively correlate with the risk of urothelial cell carcinoma and obesity, whereas the levels of plant proteins exhibit a negative correlation.

Proteins and amino acids are major biological macromolecules that serve as structural constituents, catalysts for enzymatic reactions, and energy sources. Many studies show that dietary proteins generally act as lifespan-limiting factors (Fig. 2). Deprivation of certain essential amino acids extends the lifespan of several model organisms, including *Drosophila* and mice (Fig. 2).

### The contribution of dietary proteins to animal aging

Studies in various model animals indicate a general negative correlation between the amounts of dietary proteins and lifespan. It is difficult to separate dietary proteins and carbohydrates from an essential diet. Therefore, many studies compared the effects of these two types of nutrients on aging by changing the dietary P:C ratio. Studies using Queensland fruit flies (Fanson *et al*., 2009), Mexican fruit flies (Carey *et al*., 2008), *Drosophila melanogaster* (Min & Tatar, 2006; Lee *et al*., 2008; Bruce *et al*., 2013), and field crickets (Maklakov *et al*., 2008) show that a low-protein/high-carbohydrate diet is associated with long lifespan; however, the overall caloric intake had minimal effects on lifespan (Mair *et al*., 2005). Similarly, low-protein/high-carbohydrate diets are linked to health and longevity in mice (Solon-Biet *et al*., 2014). In *Drosophila*, insulin-like peptides (*dilps*) have been shown to mediate the effects of the P:C ratio on lifespan by regulating target of brain insulin (*tobi*), which encodes an α-glucosidase (Buch *et al*., 2008). Reduced protein intake also appears to extend lifespan by inhibiting the insulin/IGF-1 or target of rapamycin (TOR) signaling pathways, which may reduce the levels of proteins with oxidative damage (Kapahi *et al*., 2004; Meissner *et al*., 2004; Sanz *et al*., 2004; Buch *et al*., 2008).

Despite a lack of direct evidence linking protein intake to human longevity, the source of dietary protein may affect human health. A large-scale, long-term study performed on European subjects indicates that high animal-protein intake positively correlates with the risk of developing urothelial cell carcinoma, whereas high plant-protein intake negatively correlates with the risk (Allen *et al*., 2013). This study also suggests that IGF-1 is a risk factor for the development of urothelial cell carcinoma in the setting of high animal-protein intake. A study of senior population reveals that subjects aged 50–65 that consumed high amount of protein had a 75% increase in overall mortality and fourfold increase in cancer-related death risk (Levine *et al*., 2014). This harmful effect seems attenuated by plant-derived protein diet. Another study that examined excess weight and obesity in Belgium indicates that the consumption of animal protein increases weight gain, whereas intake of plant protein is negatively associated with excess weight and obesity (Lin *et al*., 2011). Further, a nutritional investigation study demonstrates that a soy-based, low-calorie diet significantly reduces total serum cholesterol and body fat percentage in obese people compared with those achieved with a traditional, low-calorie diet (Liao *et al*., 2007). Although the mechanisms remain elusive, these studies reveal potential health benefits from diets that are enriched for plant proteins. Interestingly, plant proteins contain considerably lower methionines than animal proteins (McCarty *et al*., 2009), and this low methionine content may underlie the beneficial effects of dietary plant proteins (see next paragraph).

### Roles of specific amino acids in longevity

In addition to the effects of overall proteins, many studies have determined the effects of specific dietary amino acids on lifespan. Under low amino acid status, methionine restriction increases lifespan in *Drosophila* by downregulating TOR signaling (Lee *et al*., 2014). Restriction of methionine extends lifespan in a variety of rat strains with different pathological backgrounds, suggesting that methionine deficiency alters the rate of aging rather than fixing a specific pathological defect (Zimmerman *et al*., 2003). Methionine restriction significantly extends the mean and maximum lifespan of mice, even when the experiments are conducted in 12-month-old animals (Miller *et al*., 2005; Sun *et al*., 2009). Methionine-restricted mice display physiological changes, such as reduced levels of insulin, IGF-1, and glucose, similar to those observed in calorie-restricted mice. However, gene expression profiles of methionine-restricted and calorie-restricted mice do not significantly overlap. Thus, these two dietary regimens may affect longevity through partly independent pathways. Methionine restriction lengthens the lifespan of male Wistar rats and decreases the production of mitochondrial reactive oxygen species (ROS) and DNA damage (Sanz *et al*., 2006; Caro *et al*., 2009; Sanchez-Roman *et al*., 2012). Lifespan extension in *C. elegans* due to treatment with metformin, a well-known antidiabetes drug, and mutations in *metr-1*/methionine synthase is associated with decreased levels of internal methionine (Cabreiro *et al*., 2013). However, several studies suggest that methionine has a positive impact on longevity. Methionine-supplemented casein- or soy-protein diets significantly lengthen the lifespan of spontaneously hypertensive rats that are prone to developing strokes (Gilani *et al*., 2006). In addition, methionine supplementation does not shorten long lifespan in *Drosophila* with DRs but does restore fecundity (Grandison *et al*., 2009). In contrast, supplementation with all kinds of amino acids or essential amino acids suppresses DR-induced longevity. Methionine restriction also causes a slight decrease in the average lifespan but does not affect reproductive fitness in *Drosophila* (Zajitschek *et al*., 2013). Thus, other amino acids, in addition to methionine, appear to have roles in lifespan regulation. Consistent with this concept, tryptophan restriction increases the lifespan of mice (De Marte & Enesco, 1986) and Evans rats (Segall & Timiras, 1976). Further, tryptophan restriction promotes resistance to surgical stress in mouse models of ischemia–reperfusion injury (Peng *et al*., 2012).

Dietary amino acid composition affects lifespan by regulating various nutrient-sensing signaling pathways. In yeast, eIF2α kinase and GCN2, which directly bind to uncharged cognate transfer RNAs (Wek *et al*., 1995; Dong *et al*., 2000), and TOR pathway components mediate longevity by acting as cellular amino acid sensors (Gallinetti *et al*., 2013). TOR signaling is inhibited and GCN2 is activated by reduced levels of internal amino acids; this inhibits overall protein translation and increases the translation of specific proteins involved in longevity (Gallinetti *et al*., 2013). In addition, restriction of dietary tryptophan protects mice from renal and hepatic ischemic injury and reduces inflammation in a *Gcn2*-dependent manner in association with reduced serum IGF-1 (Peng *et al*., 2012). Longevity of yeast due to methionine restriction appears to be mediated by TOR signaling (Laxman *et al*., 2013) and autophagy (Sutter *et al*., 2013), a process that recycles cellular components during nutrient deprivation. The anti-aging effects of CR are largely conserved from nematodes to primates. Therefore, it is worth investigating whether the mechanisms through which amino acid restriction promotes healthy and long lifespan are also evolutionarily conserved.

## Dietary lipids exert various effects on aging

**Figure 3:**
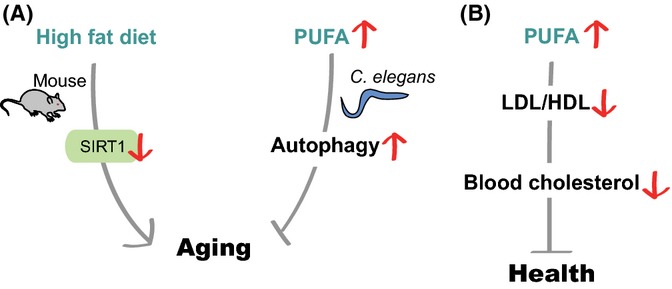
The amount and composition of dietary lipids influence organismal longevity. (A) In mice, a high-fat diet (HFD) inactivates SIRT1 and shortens lifespan. The composition of dietary fatty acids is critical for animal health. For example, ω-6 poly-unsaturated fatty acids (PUFAs) activate autophagy to promote longevity in *Caenorhabditis elegans* and probably in mammals. (B) PUFA-enriched diets decrease the ratio of low-density lipoprotein (LDL) to high-density lipoprotein (HDL); this results in reduced levels of blood cholesterol and improves health by ameliorating aging-associated diseases.

Dietary lipid components, including fatty acids, phospholipids, cholesterol, and glycerides, constitute the main structures in biological membranes. In addition, dietary lipids influence organismal physiology, including aging. A high-fat diet (HFD) is generally associated with increased mortality and increased incidence of many metabolic diseases, including type II diabetes and cardiovascular problems (Schrager *et al*., 2007; Honda *et al*., 2007) (Fig. 3). However, some specific lipids are beneficial for health and possibly longevity.

### Metabolic regulators including SIRT1 counteract the effects of HFD on metabolic dysfunction and lifespan

Genetic factors that regulate HFD-induced pathology include SIRT1 (sirtuin 1, an NAD-dependent protein deacetylase), AMPK, peroxisome proliferator-activated receptors (PPARs), sterol regulatory element-binding protein 1 (SREBP-1), carbohydrate-responsive element-binding protein (ChREBP), superoxide dismutase 3 (SOD3), cysteine-aspartate protease-1 (caspase-1), and others (Lomb *et al*., 2010; Zadra *et al*., 2010; Jeon & Osborne, 2012; Dixon *et al*., 2013; Filhoulaud *et al*., 2013; Cui *et al*., 2014; Grygiel-Gorniak, 2014). Among them, SIRT1 is one of the best-studied factors mediating the effects of HFD on metabolism and lifespan in mammals. SIRT1 acts as a key cellular sensor for nutrient availability and regulates the activities of substrate proteins (Haigis & Guarente, 2006). Upregulation of SIRT1 improves glucose tolerance and insulin sensitivity in response to a HFD (Banks *et al*., 2008; Pfluger *et al*., 2008). Conversely, white-adipose-tissue-specific SIRT1-knockout mice display metabolic dysfunctions, such as insulin resistance, increased body weight, and excess levels of fat in high-fat-feeding conditions (Chalkiadaki & Guarente, 2012). Treatment with SIRT1-activating small molecules, including resveratrol and SRT1720, prevents adverse effects of HFD on metabolism and lifespan (Baur *et al*., 2006; Lagouge *et al*., 2006; Feige *et al*., 2008; Minor *et al*., 2011; Price *et al*., 2012). Despite the controversy regarding resveratrol as a direct SIRT1 agonist (Pacholec *et al*., 2010), the beneficial effects of resveratrol and SRT1720 largely disappear in SIRT1-knockout mice (Minor *et al*., 2011; Price *et al*., 2012). Further, resveratrol or SRT1720 treatment improves mitochondrial biogenesis and function via peroxisome proliferator-activated receptor gamma coactivator-1 alpha (PGC-1α) and estrogen-related receptor alpha (ERRα) (Baur *et al*., 2006; Lagouge *et al*., 2006; Feige *et al*., 2008; Price *et al*., 2012). Thus, enhanced SIRT1 activity may improve organismal survival in the context of HFD by upregulating genes that enhance mitochondrial function and reducing excess energy storage.

### Dietary lipid composition and organismal lifespan

The composition of dietary lipid has dramatic effects on the level of blood cholesterol, which is crucial for the health of mammals. Diets enriched in unsaturated fatty acids lead to reduced blood levels of harmful low-density lipoproteins and increased levels of protective high-density lipoproteins (Mensink *et al*., 2003). Consistently, diets enriched in natural unsaturated fatty acids lower blood pressure, improve insulin sensitivity, and reduce the risks of cardiovascular and metabolic diseases (Summers *et al*., 2002; Appel *et al*., 2005). In contrast, dietary trans-fats (unsaturated fatty acids with trans-isomers) trigger inflammatory responses, which increase the risks of developing cardiovascular and metabolic diseases (Mozaffarian *et al*., 2004; Mozaffarian, 2006a,b; Riserus *et al*., 2009). Dietary saturated fatty acids are thought to be harmful to animal health, but this remains controversial (Siri-Tarino *et al*., 2010). Somewhat surprisingly, dietary cholesterol has been shown to marginally impact blood cholesterol levels (Fernandez, 2006). Overall, the composition of dietary lipid appears to be critical for blood cholesterol levels and may subsequently affect metabolic diseases and organismal lifespan.

Several studies indicate that polyunsaturated fatty acids (PUFAs) prevent aging-associated diseases and promote longevity. For example, arachidonic acids, which are omega (ω)-6 PUFAs, induce apoptosis of cancer cells (Cao *et al*., 2000). In addition, dietary arachidonic acids and eicosapentaenoic acids, which are ω-3 PUFAs, alleviate age-dependent neurodegeneration by increasing the expression of genes that are crucial for neurogenesis, neurotransmission, and neural connectivity (Das, 2008). In *C. elegans*, ω-6 PUFA feeding increases lifespan and resistance against nutrient deprivation by inducing autophagy (O'Rourke *et al*., 2013). In addition, ω-6 PUFAs activate autophagy in cultured mammalian cells, raising the possibility that similar life-extending mechanisms exist in mammals (O'Rourke *et al*., 2013).

Dietary lipids may affect mammalian health and longevity by altering the compositions of body fat and cellular membranes (Pamplona *et al*., 1998; Mitchell *et al*., 2007; Hulbert *et al*., 2008; Hulbert, 2010). Membrane PUFA levels are relatively low in the long-lived naked mole rat (*Heterocephalus glaber*) and the short-beaked echidna (*Tachyglossus aculeatus*) (Mitchell *et al*., 2007; Hulbert, 2010). This raises the possibility that membrane PUFA levels are linked to longevity. Consistent with that possibility, offspring from humans with long lifespan have low levels of PUFAs in the membranes of erythrocytes (Puca *et al*., 2008). Saturated fatty acids and monounsaturated fatty acids are generally more resistant to oxidative damage than that of PUFAs with multiple double bonds (Halliwell & Gutteridge, 1999; Hulbert *et al*., 2008). Thus, opposite from their potential role as dietary lipids, low levels of PUFAs in the membranes may be beneficial for longevity and health.

## Effects of vitamins and minerals on aging

**Figure 4:**
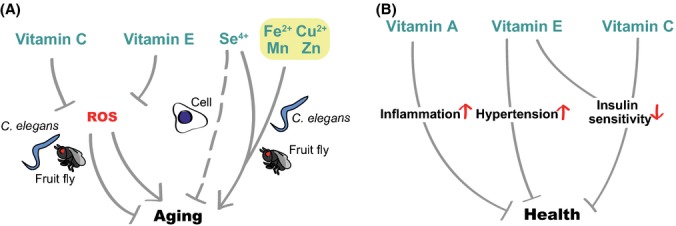
Effects of dietary vitamins and minerals on aging. (A) Vitamin C and vitamin E affect aging by acting as anti-reactive oxygen species (ROS) agents, which in turn decreases or increases lifespan in a context-dependent manner. In the case of minerals, supplementation with selenium (Se^4+^) delays cellular senescence, whereas dietary Se^4+^, Fe^2+^, Cu^2+^, Mn, and Zn confer short lifespan in *Caenorhabditis elegans* and/or fruit flies. (B) Implications for vitamins and minerals in human aging. Vitamin A increases the level of inflammation in patients with multiple sclerosis. Vitamin E increases blood pressure in patients with type II diabetes. Supplementation with vitamin E and C reduces insulin sensitivity by decreasing oxidative stress.

Although vitamins and minerals are not generally considered energy sources, these essential nutrients act as cofactors for diverse biological processes, such as mitochondrial energy metabolism and hormonal signaling (Ames *et al*., 2005). Humans cannot synthesize minerals or most vitamins; therefore, these must be supplied through dietary consumption. Deficiencies of essential vitamins and minerals can impair biological functions and promote the development of various diseases. Many studies indicate that vitamins and minerals also influence organismal lifespan (Fig. 4).

### Many vitamins and minerals influence aging by acting as antioxidants

Studies have shown that dietary vitamins increase lifespan in various organisms primarily by functioning as antioxidants. For example, vitamin E/tocopherol intake significantly increases the lifespan of rotifers, nematodes, and fruit flies (Miquel *et al*., 1982; Sawada & Enesco, 1984; Harrington & Harley, 1988; Navarro *et al*., 2005). Vitamin E also increases the replicative lifespan of cultured adrenocortical cells and protects these cells from DNA-strand breaks in peroxide-treated conditions (Hornsby & Harris, 1987). Vitamin P/hesperidin increases the lifespan of yeast by reducing ROS (Sun *et al*., 2012). Supplementation of vitamin C/ascorbic acid, a well-known antioxidant, increases the lifespan of the bean beetle *Callosobruchus maculatus* (Garg & Mahajan, 1993). Although vitamin C feeding does not change the lifespan of *D. melanogaster*, vitamin C content declines with age in flies, suggesting that decreased vitamin C may be an indicator of aging (Massie *et al*., 1991). Furthermore, diets that include vitamin C rescue the short lifespan of *wrn-1* (Werner helicase 1) mutant *C. elegans* by reducing the high levels of ROS and increasing the low levels of ATP in these mutant animals (Dallaire *et al*., 2012). Many members of the vitamin B family also lengthen the lifespan of flies, Zucker fatty rats, and *C. elegans* (Massie *et al*., 1993; Preuss *et al*., 2011; Schmeisser *et al*., 2013). For example, supplementation with vitamin B3 (nicotinic acid and nicotinamide) lengthens the lifespan of *C. elegans* through SIR-2.1, a worm homolog of SIRT1 (Schmeisser *et al*., 2013). These studies confirm the public belief that vitamins are generally beneficial for health, mostly because they moderate levels of ROS.

Although vitamins are generally considered to have beneficial effects on health, there is increasing evidence that vitamins also reduce lifespan. The antioxidant functions of vitamin C/ascorbic acid decrease the long lifespan conferred by mildly increased ROS in *C. elegans* (Schulz *et al*., 2007; Gomez-Cabrera *et al*., 2008; Yang & Hekimi, 2010). Feeding vitamin C and/or E shortens lifespan in the phlebotomine sand flies *Lutzomyia longipalpis* (Diaz-Albiter *et al*., 2011) and in wild-derived voles (Selman *et al*., 2013). In addition, vitamin C feeding reduces the enhanced mitochondrial functions caused by exercise in rats (Gomez-Cabrera *et al*., 2008). This is associated with reduced expression of PGC-1, nuclear respiratory factor 1 (NRF-1), and mitochondrial transcription factor A (mTFA), which are key transcription factors required for mitochondrial biogenesis. Consistently, vitamin C and E supplementation decreases oxidative stress but inhibits the beneficial effects of physical exercise on enhanced insulin sensitivity in humans (Ristow *et al*., 2009). Vitamin E intake causes hypertension in patients with type 2 diabetes (Ward *et al*., 2007). Moreover, a mega-dose of vitamins and minerals mildly increases human mortality (Lesperance *et al*., 2002). A meta-analysis of 385 publications indicates that overall levels of antioxidant supplementation positively correlate with mortality (Bjelakovic *et al*., 2007). In the case of multiple sclerosis, supplementation with vitamin A for 6 months increases the level of C-reactive protein (CRP), which is indicative of the level of inflammation (Jafarirad *et al*., 2013). Because moderate levels of ROS are beneficial for health and longevity (Heidler *et al*., 2010; Lee *et al*., 2010; Yang & Hekimi, 2010), antioxidant vitamins may interfere with the beneficial roles of ROS. In addition to these antioxidant vitamins, vitamin B9/folate displays a negative correlation with longevity in certain conditions, as reduced dietary vitamin B9 extends lifespan in *C. elegans* (Virk *et al*., 2012; Cabreiro *et al*., 2013). Supplementation with nicotinamide, which is one form of vitamin B3, shortens the lifespan of budding yeast by decreasing the deacetylase activity of Sir2 (Bitterman *et al*., 2002). Overall, these studies indicate that the conventional view that vitamins promote health benefits and delay aging should be modified or applied with caution.

How can we explain these differential effects of vitamin supplementation on lifespan? One plausible interpretation is hormesis, which is defined as beneficial effects of low doses of substances that are toxic at higher doses. Thus, hormetic effects of vitamins predict that high doses of vitamins have negative effects on the health and aging, while low doses are beneficial for health (Hayes, 2007). In the same context, although the amount of vitamins that are required for the proper functions of our body is relatively small, deficiency of vitamins causes diseases. The triage theory may help explain the effects of vitamin deficiency on health (Ames, 2006; McCann & Ames, 2009). According to this theory, when a micronutrient is insufficient, nature prioritizes biological functions essential for short-term survival by the expense of nonessential functions. This leads to long-term consequences that may cause age-related diseases. In any case, these possibilities are consistent with the fact that adequate amounts of vitamins are crucial for the management of health.

In comparison with vitamins, the effects of dietary minerals on aging are not as well known. Examples of the beneficial effects of minerals are rare. One example is a study showing that dietary intake of selenium (Se), an antioxidant mineral, significantly reduces DNA breakage and extends the replicative lifespan of cultured adrenocortical cells (Hornsby & Harris, 1987). However, supplementation with high doses of minerals generally decreases organismal lifespan. Supplementation with various doses of selenium (Se), iron (Fe), manganese (Mn), copper (Cu), or zinc (Zn) leads to reduced lifespan in *D. melanogaster* and *C. elegans* (Hornsby & Harris, 1987; Wang *et al*., 2007; Hu *et al*., 2008; Bahadorani *et al*., 2010; Helmcke *et al*., 2010; Bonilla *et al*., 2012; Selman *et al*., 2013). Overexpression of metal-responsive transcription factor, MTF-1, rescues the reduced lifespan of flies induced after supplementation with high, millimolar doses of metals (Bahadorani *et al*., 2010). Thus, excessive amounts of dietary minerals are generally harmful to organisms.

## Conclusions

It is well documented that CR increases lifespan in various organisms. However, CR can be difficult to execute for humans because of various reasons. Although drugs that mimic CR have been extensively sought to obtain the benefits of CR without reducing caloric intake, the effects of these drugs on human aging and health are not fully verified (Mouchiroud *et al*., 2010). Altering the amounts of each individual nutritional component in food is probably less difficult than restricting overall caloric intake, because one may not have to suffer from hunger with proper diet plans. In this review, we discussed findings that each dietary nutritional component, such as carbohydrates, proteins and amino acids, lipids, and vitamins and minerals, influences lifespan in a diverse range of model organisms. These studies raise the possibility that restriction or intake of certain types of nutrients may extend lifespan in humans as well.

There are many remaining challenges ahead in the field. First, although alteration of one nutrient can affect lifespan, this may lead to a change in the intake or processing of other nutrients in the mixture. In this regard, some of the studies on the reduction of a single nutritional component may have been misinterpreted. In addition, recent studies indicate that dietary balance among nutrients has bigger effects on aging than individual components (Lee *et al*., 2008; Skorupa *et al*., 2008; Solon-Biet *et al*., 2014). Indeed, many studies show that protein/nonprotein nutrient ratio rather than amount of proteins or calories plays key roles in the regulation of lifespan (Mair *et al*., 2005; Lee *et al*., 2008; Skorupa *et al*., 2008; Fanson *et al*., 2009; Bruce *et al*., 2013). The Nutritional Geometric Framework (NGF) is a state-space approach that represents the effects of the number and the nature of nutrient dimensions on biological responses including lifespan (Fanson *et al*., 2009; reviewed in Piper *et al*., 2011; and Tatar *et al*., 2014). NGF provides new insights into the impact of multiple nutrients on DR relative to each other. So far, this method has mostly been applied for the impact of protein intake relative to carbohydrates. Using this method, in the future, mechanisms by which different compositions of various nutrients, including lipids, amino acids, and vitamins, affect aging can be dissected better. Second, genetic factors that mediate the effects of nutritional components on aging have been mostly focused on insulin/IGF-1 signaling, TOR signaling and sirtuins, but it does not necessarily mean that these factors are the most important factors. Therefore, identification of genetic factors using unbiased methods and systems biology approaches may lead to better mechanistic insights. Third, although studies on human subjects offer invaluable information about the effects of dietary nutritional components on health and aging, more studies on primates and humans are required. For example, the effects of DR on primate longevity are controversial, perhaps due to differences in dietary nutrient composition (Colman *et al*., 2009, 2014; Mattison *et al*., 2012). In the future, it will be exciting to combine all these approaches to translate discoveries in model organisms into therapeutic applications.
